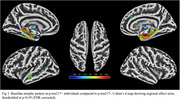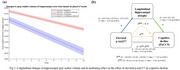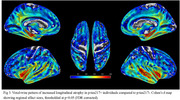# Baseline and long‐term longitudinal atrophy patterns of cognitively unimpaired individuals with elevated plasma ptau‐217

**DOI:** 10.1002/alz70856_105581

**Published:** 2026-01-08

**Authors:** Cristina Sánchez, Linda Zhang, Jesús Silva‐Rodríguez, Elizabeth Valeriano‐Lorenzo, Francisco J. López‐González, Sonia Wagner, Teodoro del Ser, Pascual Sanchez‐Juan, Michel J. Grothe

**Affiliations:** ^1^ CIEN Foundation, Reina Sofia Alzheimer Center, ISCIII, Madrid, Madrid, Spain; ^2^ CIEN Foundation, Reina Sofía Alzheimer Centre, ISCIII, Madrid, Madrid, Spain

## Abstract

**Background:**

Plasma *p*‐tau217 is an emerging biomarker for Alzheimer's disease (AD) and has been shown to be a strong predictor of cognitive decline even in cognitively unimpaired (CU) individuals. However, little is known about the association of elevated plasma *p*‐tau217 with long‐term neurodegenerative trajectories. This study aims to characterize cross‐sectional and longitudinal patterns of gray matter atrophy in CU individuals with elevated plasma *p*‐tau217 levels.

**Method:**

1030 CU older individuals (74.9±3.9 yrs; 65.1% female) from the Vallecas Project cohort at the CIEN Foundation (Madrid) were included in this study. All participants underwent blood sampling, clinical and neuropsychological evaluations, and 3T‐MRI scanning at baseline, and 863 individuals had annual longitudinal follow‐up assessments for up to ten years (average follow‐up: 5.4±2.6 yrs; for a total of 4646 MRI acquisitions). Baseline plasma *p*‐tau217 levels were measured using the LUMIPULSE platform, and individuals were classified as having elevated (*p*‐tau217+) or normal (*p*‐tau217‐) *p*‐tau217 levels based on a pre‐established threshold of 0.247‐pg/mL. T1 MRI images were processed in CAT12/SPM12 to obtain segmented gray matter maps, which were analyzed using both ROI‐based and voxel‐wise approaches. Cross‐sectional analyses employed ANCOVA models and longitudinal analyses employed linear mixed effects models. Finally, a mediation analysis was performed to determine whether plasma *p*‐tau217 affects cognitive decline through its impact on longitudinal atrophy.

**Result:**

173 participants (16.8%) were classified as *p*‐tau217+. At baseline, these individuals showed significant atrophy of the medial temporal lobe (MTL) compared to *p*‐tau217‐, particularly in the hippocampus (Fig‐1). Longitudinally, these MTL regions also showed significantly faster atrophy (*p* <0.001), which partially mediated the effect of plasma *p*‐tau217 on cognitive decline (mediated proportion: 19.4%, *p* <0.001) (Fig‐2). However, voxel‐wise longitudinal analyses also revealed a more extensive pattern of accelerated atrophy that extended beyond the MTL, additionally affecting lateral temporal areas, the insula, and the anterior and posterior cingulate cortex (Fig‐3).

**Conclusion:**

This large‐scale longitudinal imaging study demonstrates that elevated plasma *p*‐tau217 levels can identify cognitively unimpaired individuals who are on a neurodegenerative trajectory towards AD. This emphasizes the diagnostic value of plasma *p*‐tau217 for early intervention and monitoring of AD progression.